# Reply to "Female Genital Mutilation in COVID-19 Scenery"

**DOI:** 10.34172/aim.2022.103

**Published:** 2022-09-01

**Authors:** Fatemeh Shaygani

**Affiliations:** ^1^Student Research Committee, Shiraz University of Medical Sciences, Shiraz, Iran

## Author’s Reply

 I would like to thank Dr. Santos and Dr. Modesto for their interest in our paper entitled: “Eradicating female genital mutilation in Iran: actions to be done” which was recently published in the *Archives of Iranian Medicine* in November 2021.^1^

 Firstly, it should be mentioned that the solutions in our opinion survey did not consider pandemic conditions and they would be highly effective under normal, non-pandemic situations; otherwise, of course, in emergencies like this recent pandemic, when there is less access for the people, more complicated solutions need to be found and implemented.

 Moreover, it is clear that providing appropriate education for people is one of the best ways for promoting reproductive and sexual health and also eliminating female genital mutilation (FGM), which are mentioned in the Sustainable Development Goal (SDG) 5.3, but due to non-approval and non-acceptance of this statement in Iran, reaching SDG’s global goals do not seem to be possible in this country.^2^ So, we mentioned that fully localized scientific education can be more effective regarding this matter in Iran so that it can be accepted by the government and the people, and with the least opposition, it can be medically effective.

 Finally, as mentioned in your letter to the editor and according to previous studies, it is necessary to point out the great importance of appropriate training for health care staff to provide the best and high quality services to victims of this form of violence.^1,3^
